# *Tropheryma whipplei* and *Giardia intestinalis* Co-Infection: Metagenomic Analysis During Infection and the Recovery Follow-Up

**DOI:** 10.3390/idr17030062

**Published:** 2025-06-01

**Authors:** Anna Anselmo, Fabiana Rizzo, Elena Gervasi, Luca Corrent, Andrea Ciammaruconi, Silvia Fillo, Antonella Fortunato, Anna Maria Marella, Silvia Costantini, Luca Baldassari, Florigio Lista, Alessandra Ciervo

**Affiliations:** 1 Defense Institute for Biomedical Sciences, 00184 Rome, Italy; annanselm@gmail.com (A.A.); luca.corrent@gmail.com (L.C.); andrea.ciammaruconi@gmail.com (A.C.); silvia.fillo@gmail.com (S.F.); antonellafortunato75@gmail.com (A.F.) florigio.lista@esercito.difesa.it (F.L.); 2 Department of Infectious Diseases, Istituto Superiore di Sanità, 00161 Rome, Italy; fabiana.rizzo@iss.it (F.R.); annamaria.marella@iss.it (A.M.M.); silvia.costantini@iss.it (S.C.); 3 Infectious Diseases Unit, ASST Papa Giovanni XXIII, 24127 Bergamo, Italy; egervasi@asst-pg23.it; 4 Department of Clinical Sciences and Translational Medicine, University of Rome Tor Vergata, 00133 Rome, Italy; 5 National Council of Research—Institute of Electronics, Information Engineering and Telecommunications, 20133 Milan, Italy; 6 School of Medicine, University of Milano-Bicocca, 20126 Milan, Italy; l.baldassari3@campus.unimib.it

**Keywords:** Whipple’s disease (WD), next-generation sequencing (NGS), gut microbiome, bioinformatics, metagenomic analyses

## Abstract

Background: Whipple’s disease (WD) is a rare infection caused by *Tropheryma whipplei*. Diagnosis is challenging and requires a combination of several data sets, such as patient history, clinical and laboratory investigations, and endoscopy with histology analyses. While persistent diarrhea is a common symptom, WD can affect multiple organs. Case description: We present the case of a 66-year-old immunocompetent patient with WD and a history of *Helicobacter pylori* infection who developed chronic diarrhea. Colonoscopy and histopathological analysis revealed the presence of foamy macrophages with periodic acid-Schiff-positive particles. Subsequently, molecular methods confirmed the clinical WD diagnosis and metagenomic analyses further identified a co-infection with *Giardia intestinalis*. The patient fully recovered after 14 months of antibiotic therapy. During pharmacological treatment, clinical and laboratory follow-ups were conducted at 6 and 12 months, and microbiome profiles were also analyzed to identify the most abundant species in the samples. Conclusion: The metagenomic analyses showed the eradication of the two pathogens and a progressive restoration to a healthy/balanced status after antibiotic therapy.

## 1. Introduction

Whipple’s disease (WD) is caused by *Tropheryma whipplei*, a gram-positive bacterium with an environmental spreading. WD is a rare illness (3 cases per 1,000,000 population) predominantly affecting males, with a mean age at diagnosis of 55 years and with no evidence for human-to-human transmission. *T. whipplei* can be found in asymptomatic carriers and often causes self-limiting gastrointestinal symptoms. WD is a systemic disease, and relapsing or chronic infection can affect several organs, including intestinal mucosa, joints, and the central nervous system. Clinical manifestations are heterogeneous, and patients may present symptoms of diarrhea, weight loss, and arthralgias. Generally, the appropriate antibiotic therapy leads to a favorable prognosis, but if left untreated, the disease can be fatal. Genetic predisposition, individual risk factors, and immunological impairments likely contribute to disease manifestation, progression, relapses, and, in particular, cases evolving with neurological complications [1,2,3].

Several studies on familial cases showed a reduction of T-cell type 1 (Th1) and Th17 vs. an increase in Th2 immune responses. Moreover, an association with human major histocompatibility complex genes DRB1*13 and DQB1*06 has been demonstrated [4,5,6].

Due to the non-specific nature of its clinical presentation, WD is often underdiagnosed, making its identification a challenge. In patients with gastrointestinal symptoms, the diagnosis of WD is typically established by detecting foamy/amorphous macrophage positivity by periodic acid-Schiff (PAS) staining in intestinal biopsies. However, in patients with a clinical suspicion but lacking gastrointestinal symptoms, biopsies may be negative for PAS staining. In recent years, molecular diagnostic tools, including PCR and sequencing-based methods, have been developed to aid in WD diagnosis. These methods are particularly useful for laboratory diagnosis and WD confirmation as well as for challenging cases, including those with extra-intestinal involvement. Notably, PCR is the method of choice to detect *T. whipplei* DNA in cerebrospinal fluid when central nervous system involvement is suspected [7]. Since the *T. whipplei* infection may result from oral–oral or fecal–oral transmission, a co-infection with other enteric pathogens sharing the same type of transmission mode cannot be excluded. Bacterial and protozoan agents such as *Helicobacter pylori* and *Giardia* were found to be associated with WD, in particular in immunocompromised subjects [8,9,10,11].

In this study, we describe a case of a 66-year-old man affected by WD. We also performed gut microbiome analysis profiles by metagenomic next-generation sequencing (NGS) during the infection and the recovery follow-ups at 6 and 12 months. In particular, the NGS approach contributed to identifying clinically undetected *T. whipplei* and *Giardia intestinalis* co-infection. The pharmacological treatment with trimethoprim and sulfamethoxazole for 14 months was effective in the resolution of symptoms. The metagenomic analysis through NGS showed the eradication of the two pathogens and progressive restoration of their healthy/balanced status after antibiotic therapy.

## 2. Case Description

In July 2022, a 66-year-old male was admitted to the Hospital Papa Giovanni XXIII, Bergamo-Italy, with fever, loss of appetite and weight, nausea, upper abdominal pain, diarrhea, severe malabsorption syndrome, and marked microcytic anemia. He reported one year of recurrent febrile episodes, asthenia, gastrointestinal symptoms, and *H. pylori* infection treated with antibiotic therapy with bismuth subsalycilate, metronidazole, tetracycline, and proton pump inhibitors. The eradication of infection was assessed, and negative results were obtained for the *H. pylori* stool antigen performed two months after the end of treatment. The patient did not report any travel history. He lived in a rural area in South Italy as a countryman in the family field, and he reported exposure to sewer.

At admission, his blood tests showed microcytic anemia (Hb 6.6 g/dL, normal range 13 to 16 g/dL) with iron deficiency (9.0 mcg/dL, reference values 60–160 mcg/dL) and an increase of C-Reactive Protein (CRP 27 mg/dL, normal range < 0.5 mg/dL). Serum protein electrophoresis showed a slight increase in alphaglobulins (11%, normal range 2% to 5.8%) and hypoalbuminemia 2,7 g/dL (normal range > 3.5 g/dL). No abnormalities in gamma globulins were detected (Table 1). He was immunocompetent, and autoimmune factors (ANA, ENA, ANCA, anti-DNA) showed no positivity. The patient did n0t show any sign of immune system macroscopic abnormalities, and other immunological analyses were not performed. Microbiological blood cultures, including HIV, HBV, HCV, *Brucella*, *Bartonella*, *Coxiella*, and *Leishmania* serologies, were negative. Stool samples were collected for occult blood research, bacterial, *Clostridium difficile*, and parasitic studies without any findings.

The chest X-ray was normal. The abdominal CT scan showed diffuse concentric thickening of the walls of distal digiunal loops in the left iliac fossa, determining the lumen’s compression, mesenteral, retroperitoneal, and bilateral inguinal diffuse lymphadenopathies (20 × 15 mm), and significant hepatosplenomegaly. A PET scan, performed under the suspicion of lymphoproliferative disorder, showed irregular and intense uptake (SUV max 15.7) in the abdominal pelvic region, especially in the left quadrants. A diagnostic lumbar puncture was performed to investigate a potential CNS involvement. CSF blood count, glucose, and protein levels were normal. Esophagogastroduodenoscopy and colonoscopy were performed to obtain biopsy samples for laboratory tests and microbiological studies. Histological examination of duodenal biopsies revealed dilated villi and diffuse infiltration of foamy macrophages in the lamina propria. These macrophages turned positive upon periodic acid–Schiff (PAS) staining. This finding indicated a probable diagnosis of WD. Duodenal biopsies and CSF samples were sent to Istituto Superiore di Sanità, Department of Infectious Disease, Rome, Italy, for molecular investigations. According to the manufacturer’s protocol, DNA was extracted from samples by QIAamp DNA mini Kit (Qiagen, Hilden, Germany). DNA extracts were tested by Real-time PCR using whipp-frw2/whipp-rev primers specific for the *hsp65* gene target of *T. whipplei* [12]. Duodenal biopsies were positive for WD, while the CSF was negative. Positive samples were genotyped by the amplification and sequencing of the 16S-23S rRNA intergenic spacer region and domain III of the 23S rRNA genes, as previously described [13,14]. WD was diagnosed on the basis of specific Real-Time PCRs and the resulting genotype 2A.

After diagnosis (T0), the patient received initial parenteral therapy of ceftriaxone 2 g once a day for 14 days in the hospital. Within the administration of the first dose, he developed symptoms consistent with a Jarisch–Herxheimer reaction, promptly resolved without any intervention, as also reported in the literature [15]. All the other ceftriaxone infusions were well tolerated and led to no significant complications. The highly intense arthralgias (wrists, elbows and knees, and fever) were resolved with paracetamol infusion. The patient recovered rapidly with complete resolution of symptoms and quickly regained weight. After patient discharge, the oral maintenance therapy was continued with co-trimoxazole (sulfamethoxazol 800 mg + trimethoprim 160 mg) twice a day for 14 months. The long course of oral trimethoprim-sulfamethoxazole therapy was well tolerated. The patient received concomitant folic acid and iron supplementation. The renal function was stable for the entire duration of treatment, as well as the hemoglobin levels (around 13 g/dL).

The follow-up blood examination showed normal results (Table 1), and duodenal biopsies after 6 (FU1) and 12 (FU2) months were PCR-negative for WD. The first gastroscopy (FU1) showed a significant macroscopic improvement of the gastric and duodenal mucosa, but histology still revealed PAS-positive foamy macrophages. The last endoscopy (FU2) and histological exam were completely normal. The abdominal CT scan was repeated after 9 months from antibiotic therapy, showing complete resolution of all alterations previously detected. The patient was fully asymptomatic for the duration of the treatment. His blood test never showed any sign of toxicity, hemoglobin (14 g/dL) and CRP levels and kidney function were stable without any symptoms recrudescence of WD.

To further investigate the characteristics of the biopsy samples, DNA from the three duodenal biopsies (T0, FU1, and FU2) were sent to the Defense Institute Biomedical Sciences, Rome, Italy, for shotgun metagenomics by using the Illumina platform. DNA integrity for T0, FU1, and FU2 samples was evaluated with Genomic DNA ScreenTape in TapeStation Systems (Agilent Technologies, Milano, Italy). DNA concentration was assessed using a Quantus fluorometer (Promega, Milano, Italy). For each sample, 1.5 ng of genomic DNA was used to prepare sequencing libraries following the Nextera XT DNA protocol according to the manufacturer’s instructions (Illumina, San Diego, CA, USA). The library fragment size distributions were evaluated on Agilent TapeStation Systems with D5000 Screen Tape (Agilent Technologies, Milano, Italy) according to the manufacturer’s instructions. Library concentration was obtained using a Quantus fluorometer. A final library concentration of 750 pM was loaded into the NextSeq 2000 P2 cartridge (Illumina, San Diego, CA, USA) (300 cycles) and run for approximately 30 h. A separate run was performed for each DNA sample library.

The raw sequencing output obtained through the Illumina NGS of the three clinical samples was around one billion paired-end reads for each sequenced sample. All the data were analyzed using the same pipeline in order to compare the results. The bioinformatics pipeline includes the following software to cover all the steps needed to derive the taxonomic composition of the samples under analysis: FastQC (v0.12.1) for reads quality checks [16]; Fastp (v.0.23.4) for trimming and adapter removal using the following parameters: q-score ≥ 20 and reads min length = 50 [17]; Bowtie2 (v2.5.3) with the default options to reduce the number of host genome reads in the dataset [18]; Kraken2 (v.2.1.3) with the following parameters: --threads 128 --use-names --paired --report --confidence 0.2 and the PlusPF_2024065 database for taxonomic classification [19]. The Standard plus Refeq protozoa and fungi contain Refeq archea, bacterial, viral, plasmid, human, UniVec_Core, protozoa, and fungi sequences. The reads classified as microbial organisms were extracted with Kraken Tools (v1.2) [20]. The species-specific reads were then aligned to the respective reference genomes using BWA-MEM (v0.7.17) and Samtools (v1.15.1) [21,22]. After the quality check and human reads depletion, the percentage of microbial reads that could be classified was 0.5%, 1.24%, and 0.6%, considering samples T0, FU1, and FU2, respectively. Table 2 reports the taxonomic classification results at the species level, considering species with at least 100 associated reads. The data related to the five most abundant species obtained by assigned reads from Kraken2 (confidence score of 0.2) were, for the sample T0 sample, *T. whipplei*, *Cutibacterium acnes*, *G. intestinalis*, *Corynebacterium genitalium*, and *Rothia mucilaginosa*; for the FU1 sample, *C. acnes*, *Malassezia restricta*, *Moraxella osloensis*, *Staphylococcus epidermidis*, and *Prevotella intermedia*; for the FU2 sample, *C. acnes*, *Mycolicibacterium fluoranthenivorans*, *M. restricta*, *Schaalia odontolytica*, and *Solobacterium moorei* (Table 2, underlined species). Overall, the microbial biodiversity (Table 2, Others) appeared to be progressively improved in the follow-up biopsies (T0: 15.64%, FU1: 21.64%, FU2: 25.20%).

**Figure 1:**
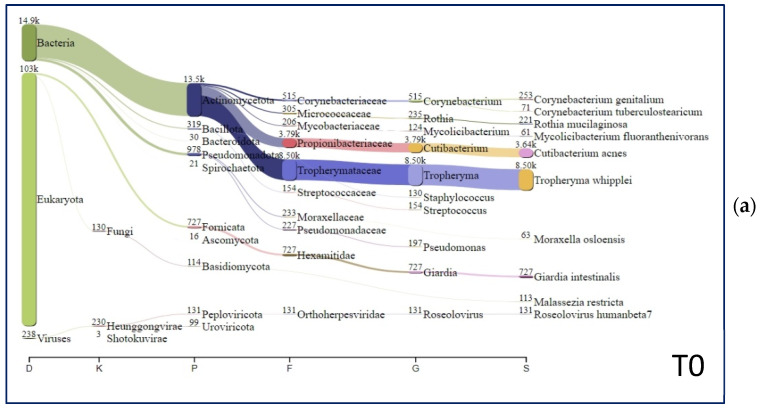

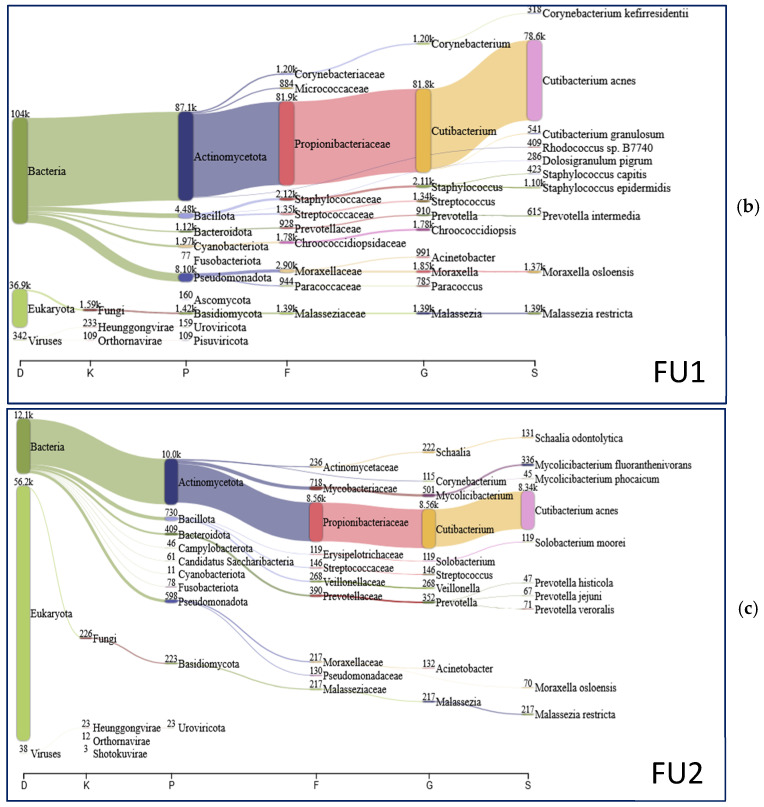
Sankey diagrams of the microorganisms found in the T0 (**a**), FU1 (**b**), and FU2 (**c**) samples. The top 10 species are represented for each sample. The most abundant species are shown in highlighted areas.

Figure 1 shows the relative abundance of the best-represented microorganisms for each sample, from the taxonomic level domain up to the species level. In sample T0, the highest proportion of classified non-human reads was represented by *T. whipplei* (8497 classified reads), followed by *C. acnes* (3639 classified reads), and the flagellated protozoan *G. intestinalis* (727 classified reads). In terms of viruses, *Roseolovirus humanbeta* 7 was identified in the T0 sample (see highlighted areas in Figure 1a). In samples FU1 and FU2, the absence of *T. whipplei* and *G. intestinalis* and a high prevalence of *C. acnes* were established (see the highlighted areas in Figure 1b,c). At the species level, the only bacteria found in all the sequenced clinical samples are represented by *C. acnes* and *M. osloensis*. Overall, it is important to highlight the preponderant presence in sample T0 of *Tropheryma whipplei* (~39% of genome coverage) and the flagellated protozoan *Giardia intestinalis* (~3% of genome coverage for each chromosome)*,* compared to the absence of *T. whipplei* and *G. intestinalis* replaced a high prevalence of *C. acnes* in the FU1 and FU2 samples. These findings were an expected output due to the pharmacological treatment.

Moreover, correlation analysis in the microbial composition changes at the three studied time points was performed by a non-parametric approach using the Spearman method. Correlations with *p* < 0.001 were considered as statistically significant. There was a positive correlation between *T. whipplei* and *C. genitalium*, both of which were present only at T0 and absent at FU1 and FU2. Other statistically significant positive correlations involved *Dolosigranulum pigrum*, *Acidovorax temperans*, *Lactobacillus iners*, *Rhodococcus* sp. *B7740*, *Lactobacillus crispatus*, and *Staphylococcus pasteuri*. All of these bacteria were present at FU1 but absent at T0 and FU2 (Table 2 and Figure 1).

## 3. Discussion

The human gut microbiome is a great example of symbiosis among various microorganisms that colonize the intestine and the host epithelial tissue. Changes in the microbiome ecosystem may run to dysbiosis, producing a decrease in microbiota biodiversity, immune response alteration, and favoring the susceptibility to infection [23,24].

Gastrointestinal symptoms, arthritis, and arthralgia are the main clinical manifestations, often treated with immunosuppressive drugs, leading to worsening or exacerbation of undiagnosed WD [25]. Most individuals acquire *T. whipplei* infection asymptomatically or show mild symptoms. However, infection susceptibility can be related to genetic factors, immunological predisposition or alteration in humoral and cellular immunity. The role of host factors is critical for the WD pathogenesis, as evidenced by a two- to threefold increase in the frequency of the HLA-B27 antigen among affected individuals [26,27].

In WD patients, the inflammatory response to the *T. whipplei* is impaired, characterized by an altered macrophage activation and dysfunction of Th1 response, favoring an activation of Th2 lymphocytes. Th2 lymphocyte polarization counteracts the Th1-mediated microbicidal activity by inducing greater expression of the anti-inflammatory cytokine IL-10. In particular, IL-10 plays a central role in immune tolerance, maintaining self-limited immune system response and tissue homeostasis. Moreover, in this scenario, the release and activation of IL-16, which is critical for monocytes and macrophage maturation/differentiation, is impaired, and the macrophages fail to undergo proper phagolysosome maturation, allowing *T. whipplei* to evade immune defenses [28,29].

Th2 drives the anti-inflammatory response, fostering the chronic infection with a consequent reduction of specific antibodies against *T. whipplei*. The immune dysregulation shapes a loop of persistent inflammation, facilitating the alteration of the intestinal barrier and spreading the pathogen through infected monocytes [29,30]. Moreover, *T. whipplei* targets immunoglobulin (Ig)A plasma cells, leading to reduced mucosal IgA levels [31,32]. This altered process may predispose WD patients to co-infections, such as *Giardia* [9].

Similarly to WD, *Giardia* infection shows a wide range of clinical manifestations, ranging from an asymptomatic status to a marked malabsorption syndrome, asthenia, abdominal pain, chronic diarrhea, and weight loss. Moreover, secretory IgA plays a critical role in immunity against *Giardia*, contributing to prolonged infection [10,33]. Studies on *T. whipplei* and *Giardia* co-infection have shown that the parasite was found either before WD diagnosis, before the antibiotic treatment or after therapeutic administration, similar to our case [8,9,34].

Since few co-infection cases have been reported and limited information is available, several hypotheses have been suggested. These include hypogammaglobulinemia status and the reduction of the macrophage CD11b expression, which is pivotal for phagocytosis [33,35,36,37]. Moreover, *Giardia* infection has been associated with a variety of gut bacteria, viruses, and parasites, including *H. pylori*. Ankarklev and colleagues, in a study conducted in Uganda, highlighted that *Giardia* and *H. pylori* co-infection was common, as it is three times more frequent than a single-species infection in asymptomatic children [38]. In particular, *H. pylori* infection impacts the biodiversity of the gut microbiome, generating dysbiosis and favoring the colonization of potential pathogens [39]. Our patient lived in a rural area and had a reported exposure to a sewer, and this might justify exposure to contaminated water and a potential co-transmission of these unusual pathogens [11]. In our case, the patient was previously treated for eradication of *H. pylori* infection, but he did not show specific symptoms for *Giardia*. However, the polymicrobial infection cannot be excluded, even before WD manifestation.

It is well known that *H. pylori* infection impacts the biodiversity of the gut microbiome, generating dysbiosis and favoring the colonization of potential pathogens [39]. In our patient, we can presume that the previous *H. pylori* infection and its eradication by antibiotic treatment may have created a gut-predisposing environment for different pathogens, particularly for *T. whipplei* and/or *G. intestinalis.* Giardia co-infection was a very interesting finding obtained retrospectively by NGS. We believe that this protozoa did not exacerbate symptoms in our patient. However, antibiotic therapy was effective in eradicating both pathogens. Co-trimoxazole is largely used in the antimicrobial therapy of diarrhea and is also effective against *Giardia* [40]. Our patient received ceftriaxone as first-line treatment for 2 weeks, followed by oral trimethoprim-sulfamethoxazole for 14 months as second-line therapy. The antibiotic treatment, generally with trimethoprim and sulfamethoxazole for 1–2 years, is crucial to avoid relapses [41]. This therapeutic approach led to the resolution of symptoms in our patient. The microbial composition analysis through NGS showed a significant shift after antibiotic therapy. *T. whipplei* and *G. intestinalis* were the most represented microorganisms in the microbiota at T0, but their complete eradication was observed at FU1 and FU2. The lack of *T. whipplei* and *G. intestinalis* at FU1 and FU2 suggested an effective therapeutic response consistent with the eradication of both species.

Moreover, the pharmacological therapy was also effective against *C. genitalium*, detected in T0 and absent in follow-up samples. From T0 to FU1 and FU2, a marked increase of *C. acnes* (T0: 22.18%, FU1: 70.50%, FU2: 66.84%) was observed, indicating a shift in the intestinal environment. *C. acnes* is a commensal bacterium that colonizes multiple tissues, including skin, oral cavity, respiratory tract, and gastrointestinal tract. Its presence in the gut may indicate a potential microbiome imbalance [42,43,44]. *M. restricta,* a commensal fungus of the skin, has recently been associated with intestinal dysbiosis and diseases such as Crohn’s disease. Sequential gut biopsies showed a progressive *M. restricta* increase (T0: 0.69%, FU1: 1.24%, FU2: 1.74%), probably reflecting an altered fungal balance, although the levels remain relatively low [45]. However, the clinical significance of these species requires further studies. Overall, the variety of microbial composition appears to improve in the follow-up biopsies (T0: 15.64%, FU1: 21.64%, FU2: 25.20%), indicating a positive signal and a progressive restoration of the healthy/balanced microbiome biodiversity [46].

Moreover, the variety of microbial species could be partially detected because they are possibly masked by the large amount of human DNA in the sequencing data. Our case presents some limitations. Sequencing technologies, such as NGS, are not widely available, and they are also expensive. In spite of the significant increase in attention and cost decrease, genome sequencing capability remains challenging. Investing limitations for facilities (reagents and instrument maintenance) is one of the main limited factors. Genome sequencing requires interdisciplinary knowledge in biology, chemistry and bioinformatics. The lack of access to genome sequencing facilities could be overcome by outsourcing, centralized sequencing facilities, international collaborations, specialized training courses, and increasing research funds. Other limitations of our study could be in the missing immunological profile of the patient that plays a role in disease pathogenesis and in the partial whole-genome sequencing of *T. whipplei* from biopsy samples to recognize antibiotic resistance genes or virulence factors or to identify in silico similarities and differences among *T. whipplei* strains.

## 4. Conclusions

The early recognition of WD disease since the onset of symptoms is crucial, and it can reduce morbidity and prevent more severe sequelae post-WD infection. Moreover, overlapping clinical symptoms with other diseases often represent a significant hurdle. For this purpose, the accurate diagnosis of WD, both with clinical and laboratory methods, is crucial and poses a diagnostic challenge. The reported case underscores the importance of a multidisciplinary approach in the care of patients affected by WD, integrating clinical evaluation, molecular diagnostics, and microbiome analysis to improve diagnostic accuracy and patient management. Our findings highlight the role of NGS as an innovative diagnostic tool for detecting co-infections that may go unnoticed with conventional methods. Indeed, in this study, NGS not only successfully confirmed *T. whipplei* infection but also identified *G. intestinalis* co-infection. Moreover, the metagenomics approach allowed monitoring of the patient’s microbiome and provided a dynamic view of microbiome variation, revealing microbial shifts related to disease progression and recovery. The eradication of pathogens, alongside the restoration of microbial diversity, suggests that microbiome-based monitoring could serve as valuable support for assessing therapeutic outcomes.

In conclusion, this case report emphasizes the critical role of NGS and whole metagenome sequencing in the management of infectious diseases through a personalized medicine approach. Further investigations are necessary to assess the long-term effects of microbiome alterations in patients with WD, refining effective therapeutic strategies. The future integration of NGS, particularly single-step detection via the shotgun metagenomics approach, into routine clinical workflows holds significant promise for enhancing diagnostic accuracy and supporting evidence-based interventions for complex infections such as WD.

## Figures and Tables

**Table 1:** Laboratory examinations at T0 (WD diagnosis), FU1 (follow-up 1), and FU2 (follow-up 2). Abnormal values are underlined.

Laboratory Examinations (Reference Range)	T0	FU1	FU2
Blood cell count, ×10^12^/L (4.3–5.9)	2.58	4.5	4.9
Hemoglobin, g/dL (13–16)	6.6	13.6	13.9
Platelet, ×10^9^/L (150–450)	171	201	183
White blood cells, ×10^9^/L (4.0–10)	5.0	6.3	6.9
Erythrocyte sedimentation rate, mm/h (0–20)	28	13	12
INR, (0.91–1.08)	0.92	0.96	0.93
Iron, mcg/dL (60–160)	9.0	70	85
Sodium, mmol/L (130–150)	135	136	135
Potassium, nmol/L (3.5–5.0)	3.6	3.7	3.9
C-Reactive protein, mg/L (<5)	27	6	2
Ferritin, mcg/L (20–270)	98	101	107
Transferrin saturation, % (15–50)	3.2	18	20
Alpha-globulins, g/dL (0.11–0.35)	0.56	0.22	0.28
Gamma-globulins, g/dL (0.58–1.75)	0.84	0.91	0.85
IgM, g/dL (0.5–2.0)	0.6	0.8	1.1
IgG, g/dL (5.3–16.5)	9.0	8.9	9.2
IgA, g/dL (0.8–5.3)	3.6	2.5	3.1
Serum creatinine, mg/dL (0.67–1.2)	0.98	0.86	0.91
Albumin, g/dL (>3.5)	2.7	4.6	4.7
Bilirubin, mg/dL (0.3–1.2)	0.3	0.6	0.8
Glutamic oxaloacetic Transaminase, IU/L (<34)	33	21	20
Glutamic pyruvic transaminase, IU/L (<49)	20	11	11
Alkaline phosphatase, IU/L (46–116)	100	97	85
Lactate dehydrogenase, IU/L (<250)	160	135	130

**Table 2:** The most abundant species identified in T0 (WD diagnosis), FU1 (follow-up 1), and FU2 (follow-up 2) samples. The five most abundant species for the three studied time points are underlined.

Species	T0 (%)	FU1 (%)	FU2 (%)
*Tropheryma whipplei*	51.79	0.00	0.00
*Cutibacterium acnes*	22.18	70.50	66.84
*Giardia intestinalis*	4.43	0.00	0.03
*Corynebacterium genitalium*	1.54	0.00	0.00
*Rothia mucilaginosa*	1.35	0.01	0.06
*Roseolovirus humanbeta7*	0.80	0.07	0.00
*Malassezia restricta*	0.69	1.24	1.74
*Moraxella osloensis*	0.38	1.23	0.56
*Mycolicibacterium fluoranthenivorans*	0.37	0.12	2.69
*Staphylococcus epidermidis*	0.32	0.99	0.10
*Staphylococcus capitis*	0.09	0.38	0.05
*Cutibacterium granulosum*	0.08	0.49	0.00
*Corynebacterium kroppenstedtii*	0.08	0.13	0.19
*Corynebacterium kefirresidentii*	0.05	0.29	0.09
*Streptococcus thermophilus*	0.05	0.10	0.03
*Schaalia odontolytica*	0.04	0.00	1.05
*Paracoccus yeei*	0.04	0.19	0.01
*Kocuria rhizophila*	0.04	0.09	0.03
*Acinetobacter johnsonii*	0.02	0.10	0.03
*Finegoldia magna*	0.01	0.09	0,00
*Streptococcus sanguinis*	0.01	0.12	0.02
*Dolosigranulum pigrum*	0.00	0.26	0.00
*Streptococcus mitis*	0.00	0.15	0.02
*Prevotella intermedia*	0.00	0.55	0.02
*Acidovorax temperans*	0.00	0.18	0.00
*Lactobacillus iners*	0.00	0.17	0.00
*Haemophilus parainfluenzae*	0.00	0.16	0.26
*Rhodococcus* sp. *B7740*	0.00	0.37	0.00
*Solobacterium moorei*	0.00	0.00	0.95
*Corynebacterium accolens*	0.00	0.17	0.02
*Lactobacillus crispatus*	0.00	0.13	0.00
*Staphylococcus pasteuri*	0.00	0.11	0.00
Others	15.64	21.64	25.20

## Data Availability

Data are contained within the article.

## Abbreviations

The following abbreviations are used in this manuscript:

CNS	Central Nervous System
CRP	C-Reactive Protein
CT	Computed Tomography
CSF	Cerebrospinal Fluid
HLA	Histocompatibility Leukocyte Antigen
Ig	Immunoglobulin
IFN-γ	Interferon-γ
IL	Interleukin
NGS	Next-Generation Sequencing
PAS	Periodic Acid-Schiff
PCR	Polymerase Chain Reaction
PET	Positron Emission Tomography
Th	T-cell type
WD	Whipple’s Diseases
